# Molecular Bases of Serotonin Reuptake Inhibitor Antidepressant-Attributed Effects in COVID-19: A New Insight on the Role of Bradykinins

**DOI:** 10.3390/jpm12091487

**Published:** 2022-09-11

**Authors:** Ahmed S. Gouda, Bruno Mégarbane

**Affiliations:** 1 National Egyptian Center for Toxicological Researches, Faculty of Medicine, University of Cairo, Cairo 11956, Egypt; 2 Department of Medical and Toxicological Critical Care, Lariboisière Hospital, Paris-Diderot University, INSERM UMRS-1144, 75010 Paris, France

**Keywords:** SSRI, COVID-19, acid sphingomyelinase, sigma-1 receptor, bradykinin, bradykinin receptor

## Abstract

Widely available effective drugs to treat coronavirus disease-2019 (COVID-19) are still limited. Various studies suggested the potential contribution of selective serotonin-reuptake inhibitor (SSRI) antidepressants to alleviate the clinical course of COVID-19. Initially, SSRI antidepressant-attributed anti-COVID-19 activity was attributed to their direct agonistic or indirect serotonin-mediated stimulation of sigma-1 receptors (Sig1-R). Thereafter, attention was drawn to the property of SSRI antidepressants to decrease ceramide production, as functional inhibitors of acid sphingomyelinase. Ceramides are cell membrane waxy lipids formed by sphingosine and a fatty acid, playing a major role in receptor signaling and infection. In COVID-19 patients, ceramide production is increased due to acid sphingomyelinase activation. Here, we aimed to review the relationships between bradykinins and the proposed pathways supporting SSRI antidepressant-attributed effectiveness in COVID-19. In COVID-19 patients, bradykinin receptor-B1 stimulation is enhanced following the downregulation of angiotensin-converting enzyme-2, which is responsible for the inactivation of des-Arg9-bradykinin, a bradykinin metabolite, contrasting with the decrease in bradykinin receptor-B2 (BDKRB2) stimulation, which results from the inhibition of cathepsin L, a kininogenase involved in bradykinin production and present at the infection site. Sig1-R stimulation modulates the inflammatory response by regulating cytokine production and counterbalances COVID-19-attributed BDKRB2 inhibition by potentiating its effects on the cytosolic calcium concentration. Moreover, the beneficial effects obtained with acid sphingomyelinase inhibition are parallel to those expected with BDKRB2 stimulation in COVID-19. Altogether, these findings suggest that one ultimate pathway of SSRI antidepressant-attributed anti-COVID-19 activity is the potentiation of BDKRB2 effects shown to be inhibited in COVID-19. In conclusion, SSRI antidepressants are able to interact positively with the pathophysiological mechanisms involved in COVID-19. However, their exact benefits in preventing morbidities or improving the outcome in COVID-19 patients remain unknown.

## 1. Introduction

Coronavirus disease-2019 (COVID-19) has spread worldwide since December 2019, causing a threatening pandemic [1]. In August 2022, about 582 million people had been infected by the severe acute respiratory syndrome coronavirus-2 (SARS-CoV-2), and 6.41 million had died. The physiopathology of COVID-19 illness is not fully understood, and its mitigation to reduce the risk of severe respiratory morbidity is still subject to intense research. Despite sustained efforts to evaluate possible therapeutic options including antiviral, anti-inflammatory, and immunomodulatory drugs, widely available effective anti-COVID-19 therapies such as ritonavir-boosted nirmatrelvir remain limited [2,3].

No connection exists between psychotropic drug treatment and an increased risk of severe COVID-19 or death. Mental illnesses in general, and particularly depression, are associated with a higher risk of dying without any reasonable clinical cause of death. They subtly reduce life expectancy. However, selective serotonin-reuptake inhibitor (SSRI) antidepressants including fluvoxamine, fluoxetine, paroxetine, sertraline, citalopram, and escitalopram are among the drugs that have been repositioned for their theoretical effectiveness in treating COVID-19 patients. Several clinical studies have been conducted including observational studies [4,5,6,7,8,9,10,11,12], prospective non-randomized trials [13,14], and randomized controlled trials [15,16], demonstrating a variable effectiveness for various tested SSRIs, especially fluvoxamine, on the clinical course of COVID-19. Table 1 presents these different studies, pointing out their flaws and limitations, which question the exact contribution of SSRIs in improving COVID-19 patient outcomes. Of note, one additional randomized study conducted in the US and Canada is still unpublished [17]. A meta-analysis (*N* = 1762 patients) including data from the two published randomized studies [15,16] and one of the two prospective cohort studies [13] did not indicate a significant influence of fluvoxamine on hospitalization, mechanical ventilation, and mortality rates [18]. By contrast, data from the three randomized studies [15,16,17] included in two other different meta-analyses (*N* = 2196 patients) suggested that fluvoxamine treatment was associated with a high probability of reducing hospitalization in COVID-19 outpatients [19,20]. Such findings encouraged recommending a prescription of fluvoxamine in COVID-19 patients without access to direct antivirals or specific monoclonal antibodies, especially in resource-limited countries. However, one should remain cautious since uncertainties about the size and relevance of the effects attributed to SSRIs including fluvoxamine persist, based on the available trials and meta-analyses [21]. Caution is even more important for critically ill COVID-19 patients with the most severe presentations and risk factors for bad outcome, due to the poorness of accurate data in this setting [22].

Different molecular mechanisms have been proposed to explain SSRI antidepressant-attributed effectiveness. Of these mechanisms, the inhibiting effects of SSRIs on acid sphingomyelinase (ASM) and the stimulating effects on sigma-1 receptors (Sig1-R) were put forward to explain the SSRI-related ability to modulate SARS-CoV-2 infectivity. Additional molecular mechanisms responsible for potential anti-inflammatory effects were suggested to counteract the deleterious effects of COVID-19.

While molecular impairments associated with COVID-19 pathogenesis are still not fully elucidated [23], major pathways have been identified, including the kinin-kallikreine system, which is claimed as playing a key role in the development of observed systemic assault and organ injuries [24,25]. Here, we aimed to review the relationships between the proposed beneficial molecular effects attributed to SSRI antidepressants in COVID-19 and the well-established disturbances in the kinin-kallikreine system.

## 2. SSRI Antidepressant-Mediated Effects on the Sigma-1 Receptor in COVID-19

### 2.1. The Non-Opioid Sigma-1 Receptor

Sig1-R, one of two sigma receptor subtypes encoded by the SIGMAR1 gene, is a chaperone transmembrane protein expressed at the endoplasmic reticulum that modulates calcium signaling through the inositol 1,4,5-trisphosphate receptor (InsP3R) [26]. Sig1-Rs form complexes with IP3R and ankyrin-B, a membrane protein implicated in in the localization and stabilization of ion transporters, ion channels, and G-proteins [27]. Sig1-R also forms complexes with other endoplasmic reticulum proteins such as the binding immunoglobulin protein (BiP) to stabilize specific signaling molecules [28]. Sig1-R agonists cause the dissociation of ankyrin-B from IP3R. This dissociation releases IP3R and activates Sig1-R, leading to IP3-dependent calcium release from the endoplasmic reticulum [29]. Sig-1R may translocate to the plasma membrane on activation, once dissociated from the endoplasmic reticulum proteins [28].

No endogenous ligand has been conclusively identified yet; however, various tryptaminergic trace amines, structurally and metabolically related to the monoamine neurotransmitters and sexual steroids including progesterone, testosterone, pregnenolone sulfate, and dehydroepiandrosterone sulfate, have been found to activate this receptor. A variety of psychotropic drugs may interact as agonists with Sig1-R including SSRI antidepressants, donepezil, and NN-dimethyltryptamine [30]. Other drugs such as haloperidol, chlorpromazine, and rimcazole present Sig1-R antagonistic properties. Interestingly, chloroquine and hydroxychloroquine, known to have potent anti-SARS-CoV-2 activities in vitro, are also considered Sig1-R ligands [30].

Sig1-R is implicated in intracellular calcium homeostasis, which is essential for the intracellular signaling of almost all cell processes, such as the proliferation, differentiation, and regulation of gene expression [29]. Besides its key role in communicating between the endoplasmic reticulum and mitochondrion for bioenergetics and cellular survival, Sig1-R has been implicated in cytoprotection, carcinogenesis, and neuroplasticity [26]. It is additionally known to regulate cytokine production, thus modulating inflammation [5,6]. Sig1-R plays an important role in the regulation of the adaptive cell stress response. Viruses use Sig1-R-mediated regulation of the stress response to promote their replication, as Sig1-R co-localizes with viral replicase proteins in endoplasmic reticulum membranes [31]. Therefore, Sig1-R agonists could exhibit beneficial anti-inflammatory, anti-oxidative, and anti-ER stress functions, improving mitochondrial biogenesis and limiting cytokine response [31,32].

### 2.2. Implication in SARS-CoV-2 Infection

Sig1-R was identified as a functional host-dependency factor in various knockdown and knockout cell systems. Sig1-R stimulation was related with the reduction in SARS-CoV-2 replication, suggesting that it should be considered as a potential key therapeutic target in COVID-19 [33]. Nevertheless, no clear correlation between the Sig1-R binding affinity and anti-SARS-CoV-2 activity was evidenced. A biobank-based approach, focused on Sig1-R gene single nucleotide polymorphism rs17775810, showed a reduced COVID-19-attributed mortality in the homozygous TT allele compared to the heterozygous TC allele and homozygous CC allele (where the major rs17775810 allele was referred to as C and the minor allele was referred to as T), suggesting a role for the Sig1-R in SARS-CoV-2 replication and supporting the possible benefits of SSRI antidepressants in COVID-19 patient outcome [34].

Surprisingly, preliminary studies suggested a possible effectiveness of Sig1-R antagonists against SARS-CoV-2 replication. COVID-19 patients treated with sigma-ligand typical antipsychotics appeared to have better outcomes in comparison to COVID-19 patients treated with atypical antipsychotics lacking anti–SARS-CoV-2 activity in vitro [33]. However, the potency of the drugs used for inhibiting SARS-CoV-2 replication (haloperidol, fuphenazine, and chlorpromazine) was not associated with their potency for antagonizing Sig1-R. By contrast, haloperidol failed to provide a clinical benefit in COVID-19 in spite of being a potent Sig1-R antagonist [33].

Sig1-R activation by small molecules has been reported to induce autophagy, an evolutionarily conserved process allowing eukaryotic cells to remove damaged parts and maintain homeostasis. Multiple viruses including Middle East respiratory syndrome-related coronavirus (MERS-CoV) and probably SARS-CoV-2 use autophagy to evade the host immune system [30]. The demonstration of Sig1-R involvement in autophagy was obtained from knocking-out experiments resulting in incomplete autophagy with a build-up of autophagosomes, whereas the transfection of Sig1-R-negative cells with a full-length Sig1-R gene copy restored the function [35]. IRSS antidepressants have been shown to induce an autophagic response in various animal models such as early brain injury after subarachnoid hemorrhage [23] and depression-related pathological alterations in astrocytes [36].

### 2.3. Interaction with the Kinin-Kallikreine System

Impairment of the kinin-kallikreine system plays a detrimental role in the pathogenesis of COVID-19 [24,25]. Stimulation of the bradykinin-receptor B1 (BDKRB1) is increased in relation to SARS-CoV-2-mediated angiotensin-converting enzyme-2 downregulation, which in turn is responsible for the inactivation of des-Arg9-bradykinin, a bradykinin metabolite that acts as the BDKRB1 ligand. By contrast, stimulation of the bradykinin-receptor B2 (BDKRB2) is decreased due to the inhibition of cathepsin L, a kininogenase involved in bradykinin production and present at the infection site.

Interestingly, bradykinin increases intracellular cytosolic calcium via the BDKRB2 [37,38]. Moreover, the direct serotonin-mediated Sig1-R stimulation has been found to have parallel effects with BDKRB2 stimulation in increasing intracellular calcium in cultured guinea-pig aortic smooth muscle cells [39]. Sig1-R-mediated ankyrin-B/IP3R-3 dissociation was also shown to result in the potentiation of a bradykinin-induced increase in cytosolic free calcium concentrations [27]. Therefore, the hypothesized deficiency in BDKRB2 stimulation in COVID-19 patients, which may result in a cytosolic calcium concentration decrease, could be mitigated through the SSRI antidepressant-mediated agonistic stimulation of Sig1-R, especially since this is accompanied by an increase in serotonin concentrations (Table 2) [40,41,42,43].

## 3. SSRI Antidepressant-Mediated Effects on the ASM/Ceramide System in COVID-19

### 3.1. The ASM/Ceramide System

ASM, a lysosomal hydrolase, catalyzes the hydrolysis of sphingomyelin to phosphorylcholine and ceramides [44]. Ceramide production on the cell surface alters its biophysical properties, forming hydrophobic gel-like ceramide-enriched membrane domains that serve to reorganize receptors and signaling molecules. Ceramides play a key role in the regulation of various cellular processes including coordinating cellular responses to extracellular stimuli and apoptosis [45]. ASM and ceramide were reported to play an important role in the receptor signaling involved in infections. The infectious agent activates ASM, releasing ceramide on the cell surface. Surface ceramide directly binds and activates a variety of enzymes and proteins that are useful for the cell defense [44,45].

### 3.2. Implication in SARS-CoV-2 Infection

Previous studies have shown that the interaction of SARS-CoV-2 spike protein with angiotensin-converting enzyme-2 (ACE2) at the cell membrane activates ASM [46]. SARS-CoV-2-induced ASM activation increases surface ceramide, resulting in the formation of a ceramide-enriched membrane that induces activated ACE2 receptors clustering. This clustering facilitates receptor signaling, resulting in SARS-CoV-2 internalization, in addition to the increased surface expression of ACE2 induced by SARS-CoV-2 infection [47]. By contrast, the inhibition of ASM or neutralization of ceramide on the cell surface prevents the infection with a SARS-CoV-2 spike, and this effect is reversed through the reconstitution of exogenous ceramide or ASM [44]. Therefore, ASM inhibitors were suggested to be able to prevent SARS-CoV-2 viral cell entry [47].

### 3.3. Interaction with the Kinin-Kallikreine System

Animal studies showed that bradykinin exerts some of its actions by modulating ASM activity. The resulting effect is that bradykinin inhibits ASM and ceramide production through specific interactions with the BDKRB2. Studies demonstrated increased sphingomyelin and decreased ceramide levels following exposure to bradykinin in addition to the reversal of these effects when using BDKRB2 but not BDKRB1 antagonists [48]. Animal studies demonstrated that BDKRB1 induction was responsible for ASM activation and ceramide increase [49].

As functional inhibitors of ASM activity (FIASMA), SSRI antidepressants inhibit ASM, leading to decreased ceramide and limiting viral cell entry [5,6]. The effects of these drugs counteract the pathophysiological effects of SARS-CoV-2 on the ASM/ceramide level. A preclinical study showed that drugs known to be FIASMAs inhibit ASM, prevent SARS-CoV-2 spike cell entry, and prevent ACE2 upregulation [44]. These effects were claimed to be one of the molecular explanations for the effectiveness of SSRI antidepressants against COVID-19 infectivity and manifestations.

**Figure 1:**
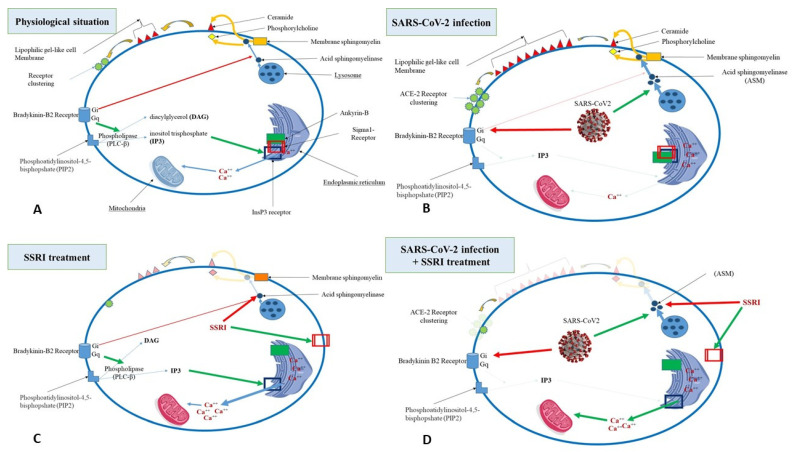
Interactions between the bradykinin receptors (BDKRB 1 and 2) and the presumed key cell molecules implicated in the beneficial effects of the selective serotonin-reuptake inhibitor (SSRI) antidepressants in severe acute respiratory syndrome coronavirus-2 (SARS-CoV-2)-infected patients. Four patterns are represented including (**A**) the baseline physiological, (**B**) the SRRI treatment, (**C**) the SARS-CoV-2 infection, and (**D**) the SARS-CoV-2 infection with the SRRI treatment situation. SARS-CoV-2 infection stimulates the release of acid sphingomyelinase (ASM) from cell membranes, leading to an increase in ceramide production and angiotensin-converting enzyme-2 (ACE2) clustering that enhances SARS-CoV-2 entry in the targeted cells. In parallel, SARS-CoV-2 infection decreases bradykinin production, thus decreasing BDKRB2 stimulation, calcium release from the endoplasmic reticulum, and adenosine triphosphate production from mitochondria. Treatment with SSRI antidepressants, known as functional inhibitors of acid sphingomyelinase, inhibits ASM release, thus decreasing ceramide production, which results in ACE2 clustering and the depression of SARS-CoV-2 cell entry. In the SARS-CoV-2-infected cells, SSRI antidepressants stimulate sigma-1 receptors, which results in the dissociation of ankyrin-B from inositol 1,4,5-trisphosphate receptors (InsP3R), thus increasing BDKRB2 stimulation, which increases the effects of inositol-3-phosphate (IP3) on the InsP3R, subsequently increasing calcium release from the endoplasmic reticulum and adenosine triphosphate production from mitochondria. Colors are lightened in the case of inhibition or reinforced in the case of stimulation. Green arrows represent stimulation, red arrows represent inhibition, blue arrows represent transfer, and yellow arrows represent a biochemical process.

Hence, SARS-CoV-2 potentiates the effect of bradykinins on the BDKRB1, leading to an increase in ceramide and the probability of viral cell entry. These observations are consistent with the theory of increased BDKRB1 and decreased BDKRB2 stimulation in COVID-19. Meanwhile, SSRIs’ effects on ASM parallel BDKRB2 effects. Figure 1 summarizes the most probable hypotheses regarding the interaction between bradykinin receptors (BDKRB 1 and 2) and the presumed key-cell molecules implicated in the beneficial effects of SSRI antidepressants in SARS-CoV-2-infected patients.

## 4. Limitations

Our minireview presents some limitations. We did not base our approach on the usual methodology that is recommended for a meta-analysis. Our work remained narrative due to the major flaws of published studies. Although the analysis of the available clinical studies clearly showed that no evidence exists to support SSRI usefulness in preventing or reducing the risks of unfavorable outcomes in COVID-19 patients, our review suggests that a molecular rationale may encourage further clinical investigations if they are based on a better methodology.

## 5. Conclusions

The potential efficacy of SSRI antidepressants as anti-COVID-19 therapy could be mediated by Sig1-R agonism and/or ASM inhibition. Additionally, these molecular effects could be explained by potentiating BDKRB2 effects consistent with the demonstrated potentiated effects of bradykinins on the anti-inflammatory BDKRB2 and their mitigated pro-inflammatory effects on the pro-inflammatory BDKRB1. These findings support once again the major role of the kinin-kallikreine system in COVID-19 pathogenesis and the complexity of interactions between the different molecular actors. Hence, the exact benefits of IRSS in preventing SARS-CoV-2 infection or improving outcomes in COVID-19 patients remain to be demonstrated.

## Figures and Tables

**Table 1:** Results of the published clinical studies on selective serotonin-reuptake inhibitor antidepressant effects in COVID-19.

No.	Study Design	Tested Molecules	Dose Regimen	Subjects	Findings	Flaws and Limitatios
[4]	Cross-sectional nationwide registry study	All psychotropic medications	Not defined	*N* = 144,321 patients	No significant association between the number of psychotropic drugs and the higher risk of severe COVID or death.	No differentiation in psychotropic drugs; heterogeneous data; no control group. Not contributive.
[5]	Retrospective observational single-center cohort study	All psychotropic medications	Not defined	*N* = 2150 patients	Independent association of previous year’s treatments with anxiolytics/hypnotics and antidepressants with lower mortality risk (HR, 0.47 and 0.43, respectively).	No differentiation in psychotropic drugs; heterogeneous data; no control group. Not contributive.
[6]	Retrospective observational multicenter cohort study	All antidepressants	Not defined	*N* = 345 treated patients among 7230 patients	Significant association between antidepressant use and reduced risk of intubation or death (HR, 0.56; CI, 0.43–0.73; *p* < 0.001). Significant association for SSRI and non-SSRI antidepressants, and for fluoxetine, paroxetine, escitalopram, venlafaxine, and mirtazapine (all *p* < 0.05).	No differentiation in antidepressants; heterogeneous data (indications, dosage, compliance, duration); no control group. Little contributive.
[7]	Retrospective observational multicenter cohort study	FIASMA medications	Not defined	*N* = 277 treated patients among 2846 patients	Significant association of FIASMA medication use with reduced likelihood of intubation or death in both crude (HR, 0.71; CI, 0.58–0.87; *p* < 0.001) and primary inverse probability weighting (HR, 0.58; CI, 0.46–0.72; *p* < 0.001) analyses. Significant association in multiple sensitivity analyses, not specific to one particular FIASMA class or medication.	No differentiation in FIASMA medications; heterogeneous data (indications, dosage, compliance, duration); no control group. Little contributive.
[8]	Multicenter retrospective cohort study with propensity score matching	Fluoxetine, fluvoxamine, or other SSRI	Not defined	*N* = 3401 patients	When compared with matched untreated control patients, reduction of mortality among patients prescribed any SSRI (RR, 0.92; CI, 0.85–0.99; *p* = 0.03); fluoxetine (RR, 0.72; CI, 0.54–0.97; *p* = 0.03); and fluoxetine or fluvoxamine (RR, 0.74; CI, 0.55–0.99; *p* = 0.04). No significant association between receiving any SSRI that is not fluoxetine or fluvoxamine and risk of death (RR, 0.92; CI, 0.84–1.00; *p* = 0.06).	Heterogeneous data (indications, dosage, compliance, duration); no control group. Little contributive.
[9]	Single-center retrospective case-control study	All antidepressants	Not defined	*N* = 34 treated vs. 368 non-treated patients	Reduction in ARDS development (*p* < 0.02) and tracheal intubation (*p* = 0.04). No significant reduction in mortality rate.	Small sample size; heterogeneous data (indications, dosage, compliance, duration); differences between the 2 groups (comorbidities and antiviral therapies) no control group. Not contributive.
[10]	Single-center retrospective case-control study	Fluvoxetine vs. observation alone	Fluoxetine 20 mg once daily	*N* = 269 patients (110 treated vs. 159 not treated)	Significant decrease in mortality (OR = 0.33; CI, 0.16–0.68; *p* = 0.002). Three cases with adverse effects requiring cessation of fluoxetine.	Small sample size; differences between the 2 groups (antiviral therapies). Little contributive.
[11]	Retrospective longitudinal, multicenter inpatient study	All psychotropic medications	Not defined	*N* = 96 patients	No tested medication was significantly associated with COVID-19 duration and severity up to the end of post-diagnosing week 3.	Limited sample size; heterogeneous data; loss of follow-up (11%); no control group. Little contributive.
[12]	Single-center retrospective case-control study	All antidepressants	Not defined	*N* = 165 patients	Protective association between antidepressant use and COVID-19 infection (OR = 0.33; CI, 0.15–0.70; *p* < 0.05). Association between lower risk of infection and fluoxetine (*p* = 0.023) and trazodone use (*p* = 0.001).	Limited sample size; no control group; no report of COVID-19 severity and outcome. Not contributive.
[13]	Prospective non-randomized comparative study	Fluvoxamine vs. observation alone	Fluvoxamine 50 mg twice daily	*N* = 98 patients. No statistical analysis	Reduction in incidence of hospitalization (0% vs. 12.5%) and persistence of residual symptoms (0% vs. 60%).	Limited sample size; possible confounders (comorbidities, comedications). Some insufficiencies.
[14]	Open-label, prospective cohort trial with matched controls	Fluvoxamine vs. observation alone	Fluvoxamine 100 mg three times daily for 15 days	*N* = 102 intensive care unit patients	No significant difference regarding the number of days on ventilator support, duration of intensive care unit, or total hospital stay. Reduction in overall mortality (58.8% vs. 76.5%, HR 0.58; CI, 0.36–0.94; *p* = 0.027).	Limited sample size; unreported matching method and adequacy; baseline between-group imbalance; analysis biases. Important insufficiencies.
[15]	Double-blind, randomized, fully remote (contactless) clinical trial	Fluvoxamine vs. placebo	Fluvoxamine 100 mg thrice daily for 15 days	*N* = 152 patients	Reduction in clinical deterioration (absolute difference, 8.7%; CI, 1.8%–16.4%, log-rank *p* = 0.009). However, study limitation due to its small sample size and short follow-up duration.	Small sample size; short follow-up; weak outcome criteria; attrition bias (20%). Interesting preliminary investigation.
[16]	Placebo-controlled, randomized, adaptive platform trial	Fluvoxamine vs. placebo	Fluvoxamine 100 mg twice daily for 10 days	*N* = 1497 patients	Reduction in the proportion of patients observed in a COVID-19 emergency setting for >6 h or transferred to a tertiary hospital due to COVID-19 (11% vs. 16%; RR, 0.68; BCI, 0.52–0.88), with a probability of superiority of 99.8% surpassing the pre-specified superiority threshold of 97.6% (risk difference, 5%). Similar findings for the modified intention-to-treat analysis (RR, 0.69; BCI, 0.53–0.90), but larger in the per-protocol analysis (RR, 0.34; BCI, 0.21–0.54). Reduction in mortality in the primary intention-to-treat analysis (OR, 0.68; CI, 0.36–1.27). Reduction in the observed mortality (OR, 0.09; CI, 0.01–0.47).	Possible confounders (comedications). Interesting investigation.

BCI, 95% Bayesian credible interval; CI, 95% confidence interval; FIASMA, functional inhibition of acid sphingomyelinase; HR, hazard ratio; OR, odds ratio; RR, relative risk; SSRI, selective serotonin-reuptake inhibitor.

**Table 2:** Reported effects on blood serotonin levels and platelet aggregation of selective serotonin-reuptake inhibitor antidepressant administration in comparison to those induced by bradykinins, observed in COVID-19 patients [34,35,36,37].

	SSRI Administration	Bradykinins	COVID-19 Patients
Blood serotonin level	Increased	ND	Decreased
Platelet aggregation	Decreased	BDKRB1: increase BDKRB2: decrease	Increased

BDKRB, bradykinin receptor-B1 or 2; ND, not determined; SSRI, selective serotonin-reuptake inhibitor antidepressant.

## Data Availability

Not applicable.
